# Selection of social comparison standards in cardiac patients with and without experienced defibrillator shock

**DOI:** 10.1038/s41598-024-51366-3

**Published:** 2024-03-06

**Authors:** Kevin Willy, Thomas Meyer, Lars Eckardt, Nexhmedin Morina

**Affiliations:** 1 Institute of Psychology, University of Münster, Münster, Germany; 2 Department of Cardiology II, University Hospital of Münster, Albert-Schweitzer-Campus 1, 48149 Münster, Germany

**Keywords:** Outcomes research, Psychology

## Abstract

Patients with an implantable cardioverter-defibrillator (ICD) often report psychological distress. Literature suggests that patients with physical disease often compare their well-being and coping to fellow patients. However, we lack knowledge on social comparison among patients with ICD. In this study, we examined psychological distress and social comparison selection in patients with (ICD+) and without experienced ICD shocks (ICD−). We theorized that relative to ICD− patients, those with ICD+ display higher levels of psychological distress and thereby compare more frequently with fellow patients with more severe disease, but better disease coping and try to identify more strongly with these standards to improve their own coping. We recruited 92 patients with (ICD+, n = 38) and without an experienced ICD shock (ICD−, n = 54), who selected one of four comparison standards varying in disease severity and coping capacity. Relative to ICD−, ICD+ patients reported higher levels of device-related distress, but there were no significant differences in anxiety, depression, or quality of life. ICD+ patients selected more often comparison standards with poor coping and, irrespective of standard choice, displayed more negative mood following comparison. Our results show that ICD+ patients tend to perform unfavorable comparisons to fellow patients, which might explain higher psychological distress and worse coping. These findings warrant further research into social comparison as a relevant coping mechanism in ICD patients.

## Introduction

In cardiovascular medicine, arrhythmias originating from the heart ventricles are often life-threatening and represent the main cause for sudden cardiac death (SCD). In patients deemed at risk for such arrhythmias or in patients already having survived it, implantable cardioverter-defibrillators (ICDs) can be implanted. ICDs can terminate arrhythmias either by overdrive pacing or by delivering a shock similarly to external cardioversion or defibrillation. In case of such an arrhythmia, patients often lose consciousness before therapy, so that the therapy itself remains pain-free. ICD implantation has been thoroughly evaluated in the last three decades and is considered an effective prevention strategy against sudden cardiac death by medical guideline recommendations^1,2^. Notably, the psychological impact of ICD implantation and its mode of action have been neglected so far.

Receiving an ICD shock can cause severe psychological distress. Indeed, ICD patients have been reported to have posttraumatic stress disorder (PTSD) rates between 8% in the primary preventive and 36% in the secondary preventive ICD population^3–6^, and ICD−related concerns have been associated with negative psychological outcomes, such as anxiety, depression and worse quality of life^7,8^. Moreover, a prospective study of 400 ICD patients found that shock experience was associated with worse mental and physical outcomes during follow-up^9^. Furthermore, perceived stress and the inability to cope with it is associated with poorer well-being^10^. Thus, ICD treatment carries a considerable psychological risk, particularly for patients who have already survived SCD or received an ICD shock (ICD+). These patients seem at higher risk for psychological distress than those without prior shock experience (ICD−). Yet, little is known about the underlying cognitive processes.

When coping with a chronic stressful situation, one highly influential factor may be social comparison, i.e., comparing with others who are in a similar situation. Indeed, it is well known that individuals frequently compare to other individuals perceived as better-off (upward), similar (lateral), or worse-off (downward) on various comparison dimensions^11,12^. One of the most widely studied key components of the social comparison process is the selection of specific comparison standards in order to seek social information. For instance, the selection of specific comparison standards may serve the self-assessment of one’s own abilities and perspectives or serve the motive of self-improvement^13^. As ICD+ might feel more severely impaired by the carriership of the ICD than ICD− patients, they might more frequently seek comparisons to and identify with standards with a severe disease course.

Concerning the impact of these comparisons, Buunk and Ybema described in their Identification/Contrast Model that focusing on similarities with an upward standard will lead to optimism about improvement resulting in positive affect; identification with a downward standard leading to the reverse effect^14,15^. Furthermore, social comparison with downward comparison is likely to induce positive emotions^16,17^. In daily life and stable, non-urgent situations, upward rather than downward comparisons are more common^12^. In cases of urgency and uncertainty, patients are in greater need for guiding information. Patients with a more severe disease might therefore prefer upward comparisons to gain diagnostic information about positive role models and to improve their own situation and their coping with the disease^18^.

Indeed, there is tentative evidence suggesting that comparisons play a critical role in physical and mental well-being^19,20^. Morina et al.^21^ found that patients seeking psychological treatment reported a higher number of upward comparisons (including social comparisons) when spontaneously explaining how they are currently doing than healthy controls. Relatedly, a recent study with 500 individuals with depressive symptoms^22^ revealed that all study participants reported to have compared their current well-being to some standard during the last three weeks. The frequency of aversive (mostly upward) comparisons was significantly related to depression and psychological well-being. Similar results were also revealed in a study with survivors of a vehicle-ramming attack^23^. This suggests that ICD patients may engage in social comparisons very frequently.

This view is supported by social comparison studies in patients with various chronic illness conditions^14,24–26^, typically finding that many patients identify with well-adjusted patients and patients with good coping, whereas they contrast to poorly adjusted ones. Arguably, identifying to upward comparisons enhances motivation to better cope with one’s own disease. In addition, Phillips and Klein^27^ point out in a review on patients with coronary heart disease (CAD), that CAD patients with a low socioeconomic status and tend to use upward comparisons leading to unfavorable psychophysiological stress responses including vasoconstriction, a greater adrenocortical stress reaction (higher cortisol levels, greater adrenocortical stress response, and higher basal heart rate) increasing the risk for occurrence and progression of CAD. For ICD patients, some findings allow an analogy in this respect, as ICD patients also have more ventricular arrhythmias and ICD shocks in situations with sympathicotonic activation (e.g. waking up in the morning^28^).

In summary, several findings suggest that ICD patients suffer from relevant psychological distress. However, we need to increase our knowledge about the extent to which ICD+ patients display higher levels of psychological distress than ICD− patients and possible reasons. Furthermore, there is lack of evidence about comparative behavior as a potential explanation for coping behavior. The present study sought to examine comparative behavior in ICD patients in terms of target selection, using a selection paradigm^25,26^. To this end, we recruited ICD patients in an outpatient ICD clinic and presented them with several questionnaires and case vignettes of fellow ICD patients. The patients were instructed to select one out of four vignettes differing in disease severity (mild vs severe) and coping (well vs poorly). In addition, we recorded data on psychological distress, mood, social comparison tendencies and coping strategies.

We hypothesized that ICD+ patients report higher levels of psychological distress (worse mood, higher anxiety, more depressive symptoms) as well as higher frequency of habitual comparative behavior related to their well-being and lower levels of quality of life than ICD− patients without experienced ICD shocks **(H1)**. Furthermore, we hypothesized that based on the existing evidence from other disease cohorts, ICD+ patients are more likely to select a standard with a severe course of disease than ICD− patients **(H2)**. Besides, we also postulated that ICD+ patients are more likely to select a standard with good/successful coping than ICD− patients **(H3)**. As ICD+ patients are supposed to suffer more severely from their illness, a confrontation with a comparison standard regardless of comparison direction should have a stronger affective impact in this group. We therefore hypothesized that ICD+ patients will report less state positive affect after reading of the case vignette than ICD− patients **(H4)**. Trying to address these hypotheses, we aim at further elucidating the psychological differences in ICD patients based on their respective clinical history and gaining insight into possible mechanisms that drive these differences. Eventually, a better understanding of psychological distress and social comparison in ICD patients can help to better identify patients in need of mental health support.

## Methods

### Participants

Between 08/2021 and 01/2022, patients were recruited for the study via the pacemaker and ICD outpatient clinic of the University Hospital Münster. The final patient sample included 92 patients in total, 38 (41%) with prior ICD shock experience and 54 (59%) without prior ICD shocks. 33 were invited to take part but did not complete a relevant number of questionnaires or did not indicate the vignette chosen and were hence excluded from the analysis. Patients with inappropriate shocks were not actively excluded, but there were no patients with inappropriate shocks in the analysis. The patients were made aware of the study through a poster placed at the entry of the outpatient clinic, which informed about the ongoing study and encouraged interested patient to ask for the questionnaire at the reception. Inclusion criteria were age ≥ 18 years, an implanted ICD with continuous follow-up (at least 3 follow-up visits) in our outpatient clinic, and information on the underlying arrhythmia leading to ICD shocks. Exclusion criteria were insufficient German language proficiency (according to the patient’s self-assessment) and severe psychiatric diagnoses being accompanied by an altered state of consciousness (i.e., patients with psychotic, bipolar, or substance use disorder). Sample characteristics can be inspected in Table 1. All participants gave written informed consent. The study was performed in accordance with the Declaration of Helsinki and approved by the local ethics committee (Ethikkommission Westfalen-Lippe, Gartenstraße 210–214, 48,147 Münster, Germany) prior to the beginning of the study (reference number: 2021-211-f-S).

**Table 1:** Baseline sample characteristics.

	Patients with experienced ICD shock (n = 38)	Patients without ICD shock (n = 54)	Difference statistic
Age in years* (*M ± SD*)*	56.4 ± 16.7	54.4 ± 16.3	*t* = − 0.581
Male sex number, percentage	29 (76%)	37 (69%)	*χ*^2^ = 0.669
Structural heart disease number, percentage	34 (90%)	48 (89%)	*χ*^2^ = 0.008
Ischemic	15 (39%)	14 (26%)	
Dilated	6 (16%)	16 (30%)	
Hypertrophic	1 (3%)	4 (7%)	
Congenital	8 (21%)	5 (9%)	
Secondary prevention	25 (66%)	20 (37%)	*χ*^2^ = 7.379**
Aborted SCD number, percentage	18 (47%)	17 (32%)	*χ*^2^ = 2.388
Heart failure symptoms (NYHA ≥ II) number, percentage	20 (53%)	20 (37%)	*χ*^2^ = 2.207
Psychiatric comorbidities	10 (26%)	5 (9%)	*χ*^2^ = 4.755*
Psychiatric medication number, percentage	8 (21%)	3 (6%)	*χ*^2^ = 5.088*

ICD, implantable cardioverter-defibrillator; SCD, sudden cardiac death; NYHA, New York Heart Association.

*p < 0.05; **p < 0.01.

### Comparison standard selection

The case vignettes presented to the patients were fictitious and in analogy to studies by Arigo et al.^25^ and Corcoran et al.^26^. There were four different vignettes to select reflecting two different levels of disease severity (mild, severe) and two types of coping (good, bad). Participants were asked to select one of the four vignettes they would like to read, based on brief descriptions. In particular the vignettes represented respectively a patient with mild disease and good coping (i.e., representing a comparison standard that is upward in terms of illness and upward in terms of coping, abbreviated UIUC standard), a patient with mild disease and bad coping (i.e. upward illness/downward coping, UIDC standard), a patient with severe disease and good coping (downward illness/upward coping, DIUC standard) and a patient with severe disease and bad coping (downward illness/downward coping, DIDC standard). The four brief descriptions were presented in a multiple-choice format on a single screen, requiring participants to select only one of them. The order of the options was randomized for each participant.

### Comparison with the selected standard

Following standard selection, participants read the respective vignette and were asked to provide self-ratings relative to the standard (relief, anxiety, happiness about personal health, fear of deterioration, coping, focusing on similarity/differences) on a six-point Likert scale (1 = *not at all*; 6 = *very much*) using 7 items in total. The focus on similarities and the contrast between the standard and themselves was assessed by analogy with Arigo et al.^25^.

### Current mood and subjective wellbeing

Current mood was assessed by a self-assessment manikin (SAM; Bradley and Lang^29^; Sierra et al.^30^) 9-point scale (1 = *very bad*; 9 = *very good*) at the beginning of the survey as well as after the case vignette with the selection of standard. Current mood SAM scores were lost for seven participants (3 in the ICD+ group) due to experimenter error. These cases were omitted from the analyses involving current mood but were retained in all other analyses.

### Depression and anxiety levels

We assessed and quantified depressive symptoms with the Patient Health Questionnaire (PHQ-9)^31^ and symptoms of anxiety with the Generalized Anxiety Disorder Scale (GAD-7)^32^. The items ask for the occurrence of the respective symptoms in the last two weeks. The PHQ-9 uses 9 items and the GAD-7 uses 7 items, all of which are scored on a 4-point Likert scale based on the frequency of symptoms. For both questionnaires good psychometric properties have been reported^31–35^.

### Quality of life

The German version of the Short Form Health Survey (SF-12) questionnaire was used to assess the overall quality of life^36,37^ and has been extensively and used in German speaking as well as international populations.^36,38^ The SF-12 makes use of 12 items, with 6 items using a 5-point Likert-scale, 2 items with a 3-point Likert scale and 4 dichotomous items. The SF-12 comprises two component scores, the Physical Component Summary (PCS) and the Mental Component Summary (MCS) with higher scores indicating a higher quality of life in the respective area. The scores range from 0 to 100 and are designed to have a mean score of 50 and a standard deviation of 10 for a representative US population. Scores > 50 therefore lie above the average health status while scores < 50 lie below^36,38^.

### Florida patients acceptance survey (FPAS)

The modified FPAS^39^ was used to assess patient acceptance of the ICD implanted. The instrument uses 15 items and comprises four underlying latent variables. These are Return to Function (RTF), Device-Related Distress (DRD), Positive Appraisal (PA), and Body Image Concerns (BIC). All the 15 items are answered on a Likert-scale reaching from 1 (*strongly disagree*) to 5 (*strongly agree*). Subscales for each of the factors as well as a total sum score were calculated, with higher scores indicating higher levels of acceptance. An exception is the DRD subscale, where scores are separately calculated to indicate higher levels of distress. The scale has been evaluated extensively^40,41^.

### Coping strategies and reaction to stress exposition

Coping behavior was assessed with the Coping Strategies Inventory Short-Form (CSI-SF)^42^. Sixteen items (5-point Likert scale) are used to assess 4 different factors of coping strategies, namely problem-focused engagement (PFE), problem-focused disengagement (PFD), emotion-focused engagement (EFE) and emotion-focused disengagement (EFD). Two overarching scales, total engagement (E) and total disengagement (D) are derived by summing the respective subscales (point range 4–20 for each subscale). The CSI-SF has been validated in various large cohorts in different languages, including German^43^.

### Frequency of upwards and downward comparisons

The frequency of different types of appetitive and aversive comparisons in relation to well-being and the engendered affect were assessed with the Comparison Standards Scale—Well-being (CSS-W)^22^. The CSS-W uses 14 items to assess comparative thoughts in relation to the current well-being over the last three weeks by asking about the frequency of comparisons to different standards on a 6-point Likert scale (ranging from 0 to 5). The assessed types of comparison are social, temporal, counterfactual, and criteria-based. Seven items address appetitive comparisons and seven items address aversive comparisons. The two subscales are summarized separately using sum scores, with higher scores indicating more frequent comparison. Initial results suggest the CSS-W to be a valid assessment tool that relates significantly to mental health complaints^22^.

### Procedure

**Figure 1:**
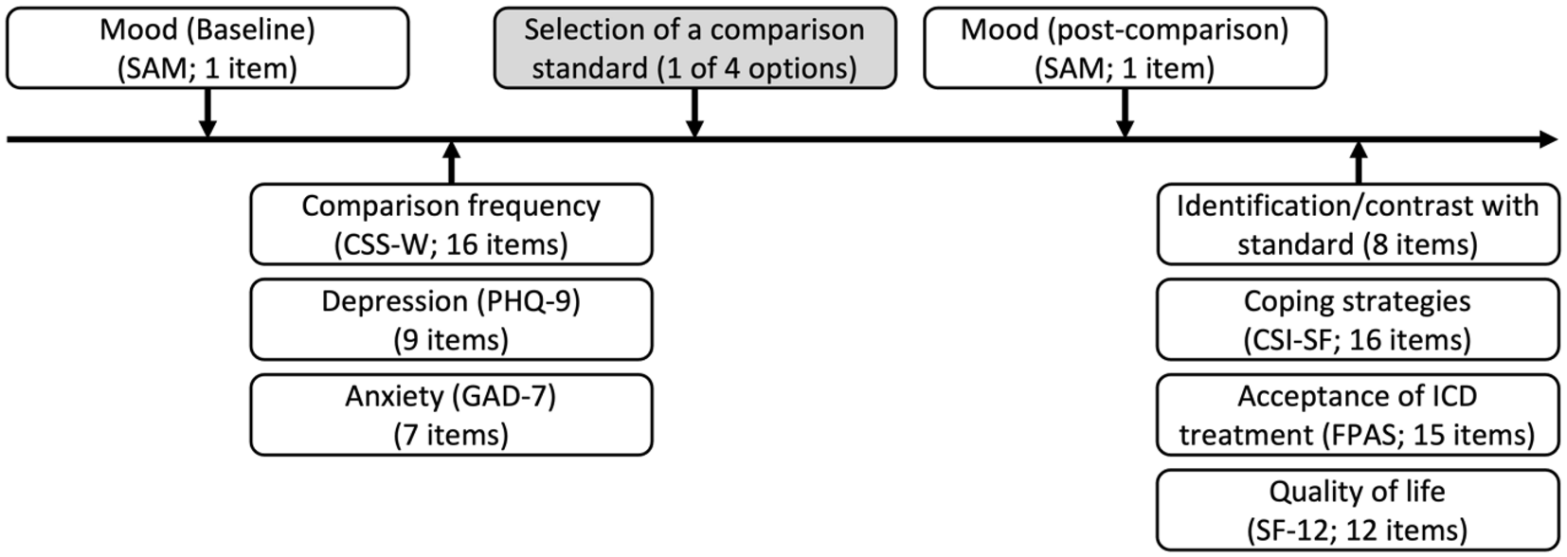
Timeline of the present study.

Participants were asked for their participation in the study during a routine visit at our outpatient ICD clinic. After receiving information about course and aim of the study and giving informed consent, participants completed all measures (for an overview, see Fig. 1). Participants were instructed to select one of four case vignettes they were most interested in reading. Afterwards they filled out the questionnaires in the order mentioned above and were thanked and debriefed. They did not receive compensation for completing the study. For overview of the order of measures see Fig. 1. In brief, participants were presented the SAM, CSS-W, PHQ-9 and GAD-7 before choosing the respective case vignette. Afterwards, they were presented with SAM, question regarding identification and contrast with the presented vignette, CSI-SF, FPAS and SF-12. All questionnaires were administered in pen-and paper format. The results of the questionnaires were complemented with medical data of the patients obtained from the patient data management system of our clinic.

#### Statistical analyses

Between-group effects, mainly focusing on mean or proportion differences between ICD+ and ICD− (cf. H1–H4), were analyzed using independent-samples *t*-tests and analyses of (co-)variance (ANOVA) to be complemented with post-hoc t-tests in case of significant interactions, and chi-squared tests. When the assumption of equality of variances was violated (i.e., significant Levene’s test for equality of variances), we used *t*-tests with Satterthwaite approximation. Moderate violations of the normality assumptions were tolerated (e.g., for several variables, significant Shapiro–Wilk tests indicated deviations from normality, whereby skewness or kurtosis values were close to ± 1), as these typically do not inflate Type 1 error rate in ANOVA. Nonetheless, we complemented the respective analyses with non-parametric Mann–Whitney U-tests and Quade’s tests. Since none of these tests yielded different results (in terms of significance levels), they are not reported below. Alpha was set at 0.05 (two-tailed) for all analyses. All statistical tests were performed using IBM® SPSS Version 28.

## Results

### Differences in psychological distress between ICD+ and ICD− patients

Anxious as well as depressive symptoms were descriptively more common in the ICD+ group (GAD-7: M = 6.4, SD = 5.4; PHQ-9: M = 8.0, SD = 5.7) than in the ICD− group (GAD-7: M = 4.7, SD = 4.9; PHQ-9: M = 6.3, SD = 5.5), but the difference was not statistically significant (respectively *t* [89] = − 1.59, *p* = 0.115, *d* = − 0.34, and *t* [90] = -1.50, *p* = 0.138, *d* = -0.32).

### Habitual comparison frequency

There was no difference in comparison frequency between the two patient groups. While ICD+ patients numerically selected aversive (i.e., mostly upward) comparisons more often (*M* = 11.9, *SD* = 6.9) than ICD− patients (*M* = 10.7, *SD* = 7.9), the difference was not significant, *t* (90) = − 0.780, *p* = 0.437, *d* = − 0.165. Concerning the choice of appetitive comparison both groups did also not differ significantly (ICD+: *M* = 8.9, *SD* = 5.5; ICD−: *M* = 8.3, *SD* = 5.9), t (90) = -0.529, *p* = 0.598, *d* = − 0.112). In terms of the absolute number of patients, 97.4% of ICD+ patients reported at least one type of aversive comparison, whereas 94.7% reported at least one type of appetitive comparison. Similarly, 88.9% of the ICD− patients reported at least one type of aversive comparison and the same percentage of patients reported at least one type of appetitive comparison.

With respect to social comparison frequency only, 76.3% of the ICD+ patients reported at least one aversive comparison and 81.6% of the ICD+ patients reported at least one appetitive comparison. On the other hand, 61.1% of the ICD− patients reported at least one aversive comparison and 66.7% of the ICD− patients reported at least one appetitive comparison. Overall, the mean of aversive (i.e., upward) social comparisons in ICD+ patients was somewhat higher (*M* = 3.1, *SD* = 2.7) than in ICD− patients (*M* = 2.6, *SD* = 2.7), yet, the difference was not significant, *t* (90) = − 0.790, *p* = 0.432, *d* = − 0.167. Likewise, the mean of appetitive social comparisons in ICD+ patients (*M* = 3.4, *SD* = 2.7) was not significantly higher than in ICD− patients (*M* = 3.2, *SD* = 2.9; *t* (90) = − 0.424, *p* = 0.673, *d* = − 0.090).

### ICD device acceptance

Concerning overall ICD acceptance, ICD+ patients reported significantly lower device acceptance (*M* = 61.0, *SD* = 10.6) than ICD− patients (*M* = 65.4, *SD* = 8.5), *t* (86) = 2.161, *p* = 0.033, *d* = 0.467. This difference appeared to be driven by higher levels of device distress in the ICD+ group (*M* = 9.84, *SD* = 4.47) than in the ICD− group (*M* = 7.59, *SD* = 2.97), *t* (86) = − 2.837, *p* = 0.010 (equal variances not assumed), *d* = − 0.613, whereas there were no group differences on the other subscales, *p*s > 0.12, *d*s < 0.34. Regarding quality of life, there were no significant differences with descriptively lower SF-12 scores in the ICD+ group for the PCS score (*M* = 43.54, *SD* = 10.4), compared to the ICD− group (*M* = 40.97, *SD* = 10.96), *t* (90) = 1.122, *p* = 0.265) as well as for the MCS score (ICD+ group: *M* = 50.71, *SD* = 11.14; ICD− group: *M* = 46.27, *SD* = 12.22, *t*(90) = 1.733, *p* = 0.080).

### Coping strategies

Concerning coping with the condition, the groups displayed similar total engagement scores on the CSI-SF (ICD+: *M* = 21.2, SD = 6.5; ICD−: *M* = 21.6, *SD* = 7.8), *t* (90) = 0.254, *p* = 0.800, *d* = 0.054. ICD+ patients tended to have higher total disengagement scores (ICD+: *M* = 16.9, SD = 7.1; ICD−: *M* = 14.0, *SD* = 7.3), *t* (90) = − 1.873, *p* = 0.064, *d* = 0.396). There were no group differences on any of the more fine-grained CSI-SF subscales, all *p*s > 0.09.

### Standard selection

Chi-squared test on the proportion of ICD+ and ICD− patients selecting the four comparison standards (severity: high/low; coping: good/bad) revealed a significant group difference (χ^2^ = 9.973, df = 3, *p* = 0.019). The respective choice frequencies are displayed in Table 2.

**Table 2:** Crosstable for the relation of prior experience of ICD shock and comparison standard selected.

		ICD group					
		ICD−		ICD+		Total	
		N	%	N	%	N	%
Standard selected	UIUC	33	61.1%	14	36.8%	47	51.1%
	UIDC	0	0.0%	2	5.3%	2	2.2%
	DIDC	3	5.6%	8	21.1%	11	12.0%
	DIUC	18	33.3%	14	36.8%	32	34.8%
Total		54	100.0%	38	100.0%	92	100.0%

The difference between the selected standard in both ICD shock groups was significant (χ^2^ = 9.973, df = 3, *p* = .019).

ICD, implantable cardioverter-defibrillator; UI, upward illness; DI, downward illness; UC, upward coping; DC, downward coping.

### Mood following standard selection

To analyze the impact of comparison standard choice, we treated the chosen levels of severity and coping as between-subjects in the following analyses. Note that severity and coping were analyzed separately because cell sizes were too small to enter both factors into the same model (see Tables 2, 3, 4).

**Table 3:** Crosstable for the relation of prior ICD shock experience and disease severity of the selected standard.

		Patient group					
		ICD−		ICD+		Total	
		N	%	N	%	N	%
Disease severity	Mild	33	61.1%	16	42.1%	49	53.3%
	Severe	21	38.9%	22	57.9%	43	46.7%
Total		54	100.0%	38	100.0%	92	100.0%

The difference between the selected standard in terms of disease severity in both ICD shock groups was not statistically significant (χ^2^ = 3.236, df = 1, *p* = .072).

ICD, implantable cardioverter-defibrillator.

**Table 4:** Crosstable for the relation of prior experience of ICD shock and the coping performance presented in the standard selected.

		Patient group					
		ICD−		ICD+		Total	
		N	%	N	%	N	%
Coping	Good	51	94.4%	28	73.7%	79	85.9%
	Bad	3	5.6%	10	26.3%	13	14.1%
Total		54	100.0%	38	100.0%	92	100.0%

The difference between the selected standard in terms of coping performance in both ICD shock groups was significant (χ^2^ = 7.922, df = 1, *p* = .005).

ICD, implantable cardioverter-defibrillator.

Baseline mood (SAM rating) did not differ between patient groups (*p* = 0.509) and was entered as a covariate in a 2 (patient group: ICD+, ICD−) × 2 (comparator severity: mild, severe) ANCOVA. This revealed that mood following standard selection was significantly worse in ICD+ patients (covariate-adjusted *M* = 5.8, *SE* = 0.3) than in ICD− patients (covariate-adjusted *M* = 6.5, *SE* = 0.2), *F* (1,80) = 4.488, *p* = 0.037, ηp^2^ = 0.053). The choice of standard concerning disease severity had no main effect on mood, F (1,80) = 0.004, *p* = 0.948, ηp^2^ < 0.001, and there was no interaction, F (1,80) = 0.374, *p* = 0.543, ηp^2^ = 0.005 **(H4)**.

However, the ANCOVA with comparator coping as a factor indicated a large main effect of comparator coping on mood, F (1,80) = 35.228, *p* < 0.001, ηp^2^ = 0.213), which did not interact with patient group, F (1,80) = 0.036, *p* = 0.851, ηp^2^ < 0.001. Respondents selecting bad coping presented worse mood (covariate-adjusted *M* = 4.4, *SE* = 0.4) than respondents selecting good coping (covariate-adjusted *M* = 6.5, *SE* = 0.2) **(H4)**.

### Identification and contrast with standards

A 2 (patient group: ICD+, ICD−) × 2 (comparator severity: mild, severe) ANOVA on self-reported identification with the selected standard did not reveal a difference between the ICD+ and the ICD− group, *F* (1,88) = 2.597, *p* = 0.111, ηp^2^ = 0.029 (across groups estimated *M* = 3.8, *SE* = 0.3), and there were no effects involving comparator severity (*p*s > 0.252). A similar ANOVA on self-reported contrast revealed no significant difference between patient groups (across groups estimated *M* = 7.0, *SE* = 0.4), *F* (1,88) = 3.064, *p* = 0.084, ηp^2^ = 0.034, and no effects involving comparator severity (*p*s > 0.363).

Meanwhile, the 2 (patient group) × 2 (comparator coping: good, bad) ANOVA revealed that patients who selected poor coping showed higher identification with the selected standard (*M* = 5.4, *SE* = 0.8) than those selecting good coping (*M* = 3.4, *SE* = 0.3), (F [1,88] = 6.040, *p* = 0.016). Comparator coping did not interact with patient group (*p* = 0.812). A similar ANOVA on self-reported contrast to the standard showed no effects involving comparator coping (*ps* > 0.619).

## Discussion

The current study aimed to explore psychological well-being and coping behavior as well as antecedents and consequences of social comparison behavior among ICD patients with and without prior ICD shock experience. The main results are as follows. First, there were no significant differences between the two patient groups concerning anxiety, depression and quality of life. Similarly, there were no significant differences in coping behavior and habitual comparison frequencies. However, ICD+ patients had lower device acceptance due to higher device-related distress (cf. **H1**). Second, ICD+ patients did not differ from ICD− patients concerning the selected standard in terms of disease severity but only in terms of disease coping. That is, although ICD+ patients chose a severe course more often and ICD− patients chose a mild course more often (cf. **H2**). Meanwhile, ICD+ patients selected standards with bad coping more often than ICD− patients (**H3**). Finally, despite comparable mood levels at the beginning of the study, ICD+ patients displayed significantly worsened mood after presentation of the case vignette than ICD− patients, irrespective of their comparison choice (**H4**).

Arigo et al. reported that patients with a more severe course of a type 2 diabetes had worse quality of life and higher levels of depression^44^. In comparison, we only found trends towards worse psychological health in ICD+ patients who also have a physically more severe disease in comparison to ICD− patients (**H1**). This might be influenced by the fact that the modifiability of the disease course is different in diabetes compared to ICD patients. Patients with diabetes can directly impact measurable surrogate parameters of disease severity such as HbA1c by their own behavior and therapy adherence. ICD patients, however, have less influence on the occurrence of arrhythmias and ICD shocks and might therefore experience less self-efficacy, which is known to be closely associated with depression and anxiety^45–47^. Self-efficacy has been shown to be a relevant mechanism in self-management and quality of life in different patient groups^48^. Thus, the role of perceived self-efficacy in ICD patients in well-being and coping behavior warrants further examination.

We found subtle differences in the choices of comparison standards. However, these were not entirely as predicted: The results did not support the prediction that ICD+ patients would select a standard with a higher disease severity more often (**H2**), although a trend in this direction was present in the data. Our hypothesis was founded on results from results from Arigo et al.^25^ who reported patients with type 2 diabetes commonly selected a better-off standard and justified it by the need for more information about successful coping. The results are in line with the Identification/Contrast Model of comparison^15^ that proposes that a downward comparison with greater identification leads to worse quality of life and less motivation, whereas identification with an upward standard increases quality of life and motivation. In our study, we found that ICD+ patients identified more strongly with the worse-off standards in line with results from Arigo et al.^44^ and selected standards with bad coping more often.

Against our expectation that ICD+ patients would select a standard with good coping more frequently than ICD− patients (**H3**), we found evidence for the opposite pattern. Poor psychological health and low self-efficacy might therefore play a role in selection standards with poor rather than good coping. The role of the ICD shock status as a relevant feature differentiating between upward and downward comparing patients is illustrated by the interesting finding that there was no relevant influence of the patient history leading to ICD implantation (primary vs. secondary prevention, aborted SCD), but only for the course of disease after ICD implantation on psychological distress. This should draw further attention to shock status as a distinctive feature in ICD patients in future studies. Practical implications should therefore contain a candid screening for common mental health complaints in this population. For example, the Patient Health Questionnaire represents a time efficient screening self-report for common mental health disorders and the average time required of the physician to review the questions is less than two minutes^31,32^. Furthermore, patients reporting mental health complaints should be encouraged to seek professional mental health. Importantly, the first period after implantation as well as first treatment of an arrhythmia by the ICD are probably the most vulnerable phases for patients and therefore demand more attention from the medical personnel.

Concerning the patients’ mood and the influence of the case vignette presented, our study results align with prior studies such with Corcoran et al.^26^, who reported a strong mood effect of the presented case vignette in women with breast cancer regardless of identification or contrast effects and regardless of the disease course. In our study, the influence of social comparison on mood was more negative in ICD+ than in ICD− patients, and thus, depended on disease severity (**H4**). This might possibly be explained by the fact, that ICD+ patients have already experienced arrhythmias and ICD shocks first-hand, whereas ICD− patients have not. Accordingly, a case vignette of a fellow patient with experienced arrhythmias and ICD shocks can provoke concrete negative memories only in the ICD+. Additional evidence from Arigo et al.^44^ illustrated that the selection of the worse-off standards worsened mood and stress levels and reduced self-care motivation. In our study, ICD+ patients had more negative mood following comparison selection than ICD− patients, irrespective of the specific selected standard. This might be due to the heterogeneity regarding age, disease severity and awareness of the ICD as a life-saving device but also a potential source of pain and distress (Supplementary Information).

## Limitations

Our study has some limitations worth to be mentioned. Comparison selection in a laboratory setting might not reflect comparison selection in real life, which further increased by the fact that the number of patients in our study was rather small, yet comparable to similar studies^25,26^. Furthermore, case vignettes varied in course of disease and coping success but did not mention information about prognosis or further medical measures exceeding the ICD performance and ICD perception. To exclude possible manipulation of identification or contrasting with the standard presented we left out information such as age, underlying heart disease and medication. However, identification or contrasting might be stronger in a more detailed description of the case vignette (e.g., younger patients reading about a young patients)^13^, which might be addressed in future studies. Furthermore, the generalizability might be hampered by the rather homogenic study population, in which most of the patients were of Caucasian origin with a high socioecomonic status. As there were for example no African-American, Latin American and Asian participants included, cultural influences and different role perceptions are not represented in the results of the study. Finally, replication in studies with larger samples is needed.

## Conclusions

Taken together, our results indicate that social comparison selection in ICD patients depends on whether they have received an ICD shock or not. ICD+ patients selected more often unfavorable social comparisons and those selecting a comparator with poor coping identified more strongly with this comparator. These social comparison strategies might negatively influence disease coping and psychological well-being, including anxiety and depressive symptoms. The results highlight the need to identify mental health complaints and coping difficulties that ICD patients might have. Future studies need to examine the role of social comparison of patients with cardiac diseases and how dysfunctional social comparison might be reduced by psychological interventions. Furthermore, implementation of short screening methods for mental health complaints in ICD patients in clinical settings may help detect patients in need of psychological treatment.

### Supplementary Information

Supplementary Information.

## Data Availability

Data is fully available upon reasonable request from the corresponding author.
