# Supplementary material for: Selection of social comparison standards in cardiac patients with and without experienced defibrillator shock
Source: Sci Rep. 2024 Mar 6;14:5551. doi: 10.1038/s41598-024-51366-3 (PMC10917798; doi:10.1038/s41598-024-51366-3)
Supplement: Supplementary file 1 — Supplementary Information. [file 41598_2024_51366_MOESM1_ESM.docx]

**DIDC Target:**

**Fallvignette**

Mein Hausarzt hat mich wegen einer Herzerkrankung an einen Kardiologen überwiesen, der diesen Verdacht bestätigen konnte und der mich dann an ein Krankenhaus weiter überwiesen hat. Dort wurde mir aufgrund der Diagnose ein Defibrillator implantiert. Ich habe mich früher nie länger niedergeschlagen gefühlt, aber die Defibrillatorimplantation nimmt mich immer noch ziemlich mit. Bereits nach kurzer Zeit hat der Defibrillator das erste Mal einen Schock abgegeben, weil ich eine Herzrhythmusstörung hatte. Seitdem hat mein Kardiologe mir Medikamente verschrieben, die die Rhythmusstörung unterdrücken sollen. Die Medikamente erschöpfen mich sehr und obwohl ich sie einnehme, hat es seit der Implantation auch noch einen zweiten Schock gegeben, sodass ich nicht weiß, ob sie mir wirklich helfen. Ich bin durch die Medikamente oft müde und fühle mich schlecht. Ich bemerke oft Herzstolpern, welches mir Angst macht und mich nervös macht. Ich bin in regelmäßiger Kontrolle bei meinem Kardiologen, der mich bestätigt, dass das Gerät fehlerfrei funktioniert, was mich aber auch nicht beruhigt. Ich weine viel mehr als vor der Implantation und mache mir Sorgen, dass der Defibrillator großen Einfluss auf mein weiteres Leben und die Beziehung zu meinen Mitmenschen hat.

**DIUC Target:**

**Fallvignette**

Mein Hausarzt hat mich wegen einer Herzerkrankung an einen Kardiologen überwiesen, der diesen Verdacht bestätigen konnte und der mich dann an ein Krankenhaus weiter überwiesen hat. Dort wurde mir aufgrund der Diagnose ein Defibrillator implantiert. Ich habe mich früher nie länger niedergeschlagen gefühlt, aber die Defibrillatorimplantation hat mich anfangs ziemlich mitgenommen. Inzwischen fühle ich mich wieder deutlich besser. Bereits nach kurzer Zeit hat der Defibrillator das erste Mal einen Schock abgegeben, weil ich eine Herzrhythmusstörung hatte. Seitdem hat mein Kardiologe mir Medikamente verschrieben, die die Rhythmusstörung unterdrücken sollen. Die Medikamente machen ein wenig müde, aber man gewöhnt sich schnell daran. Seitdem ich sie einnehme, hat es noch einen zweiten Schock gegeben, ich glaube aber daran, dass die Medikamente mich vor weiteren Herzrhythmusstörungen schützen. Ich bekomme manchmal kurzes Herzstolpern, bin aber nicht besorgt, weil ich weiß, wie der Defibrillator funktioniert und programmiert ist. Ich bin in regelmäßiger Kontrolle bei meinem Kardiologen, der mich bestätigt, dass das Gerät fehlerfrei funktioniert. Insgesamt hat mir der Defibrillator bisher zwei Mal das Leben gerettet und ich bin froh, dass ich ihn habe. Der Defibrillator steht mir in meinem Leben nicht im Weg.

**UIDC Target:**

**Fallvignette**

Mein Hausarzt hat mich wegen einer Herzerkrankung an einen Kardiologen überwiesen, der diesen Verdacht bestätigen konnte und der mich dann an ein Krankenhaus weiter überwiesen hat. Dort wurde mir aufgrund der Diagnose ein Defibrillator implantiert. Ich habe mich früher nie länger niedergeschlagen gefühlt, aber die Defibrillatorimplantation nimmt mich immer noch ziemlich mit. Ich bin in enger Betreuung durch meinen Kardiologen, der mir gesagt hat, dass ich vorerst keine Medikamente für das Herz nehmen muss und mein Leben normal weiterleben kann, da das Risiko für eine Schockabgabe durch den Defibrillator bei mir sehr gering ist. Trotzdem bemerke ich oft Herzstolpern, welches mir Angst macht und mich nervös macht. Oft glaube ich, dass ich bald einen Schock durch den Defibrillator bekomme, obwohl dies noch nie passiert ist. Ich bin in regelmäßiger Kontrolle bei meinem Kardiologen, der mich bestätigt, dass das Gerät fehlerfrei funktioniert, was mich aber auch nicht beruhigt. Ich weine viel mehr als vor der Implantation und mache mir Sorgen, dass der Defibrillator großen Einfluss auf mein weiteres Leben und die Beziehung zu meinen Mitmenschen hat.

**UIUC Target:**

**Fallvignette**

Mein Hausarzt hat mich wegen einer Herzerkrankung an einen Kardiologen überwiesen, der diesen Verdacht bestätigen konnte und der mich dann an ein Krankenhaus weiter überwiesen hat. Dort wurde mir aufgrund der Diagnose ein Defibrillator implantiert. Ich habe mich früher nie länger niedergeschlagen gefühlt, aber die Defibrillatorimplantation hat mich anfangs ziemlich mitgenommen. Inzwischen fühle ich mich wieder deutlich besser. Ich bin in enger Betreuung durch meinen Kardiologen, der mir gesagt hat, dass ich vorerst keine Medikamente für das Herz nehmen muss und mein Leben normal weiterleben kann, da das Risiko für eine Schockabgabe durch den Defibrillator bei mir sehr gering ist. Ich habe mich inzwischen gut an den Defibrillator gewöhnt. Ich bekomme manchmal kurzes Herzstolpern, bin aber nicht besorgt, weil ich weiß, wie der Defibrillator funktioniert und programmiert ist. Er musste auch bisher nie aktiv werden und in den Kontrollen, zu denen ich regelmäßig gehe, sagen mir die Ärzte, dass er fehlerfrei funktioniert. Ich bin froh, dass er mich beschützt. Insgesamt steht mir den Defibrillator in meinem Leben nicht im Weg.
